# Supplementary material for: Specific microRNA Signature Kinetics in Porphyromonas gingivalis-Induced Periodontitis
Source: Int J Mol Sci. 2023 Jan 24;24(3):2327. doi: 10.3390/ijms24032327 (PMC9916963; doi:10.3390/ijms24032327)
Supplement: Supplementary file 1 [file ijms-24-02327-s001.zip › ijms-2147625-supplementary.pdf]

International Journal of
Molecular Sciences

MDPI

Article

Specific microRNA Signature Kinetics in *Porphyromonas gingivalis*-Induced Periodontitis

Chairmandurai Aravindraja [1,†], Krishna Mukesh Vekariya [1], Ruben Botello-Escalante [1], Shaik O. Rahaman [2], Edward K. L. Chan [3] and Lakshmyya Kesavalu [1,3*]

[1] Department of Periodontology, College of Dentistry, University of Florida, Gainesville, FL 32610, USA; Aravindraja.Chairmandurai@neurology.ufl.edu (C.A.); kvekariya@ufl.edu (K.M.V.); rbotelloescalant@ufl.edu (R.B.-E.)
[2] Department of Nutrition and Food Science, University of Maryland, College Park, MD 20742, USA; srahaman@umd.edu
[3] Department of Oral Biology, College of Dentistry, University of Florida, Gainesville, FL 32610, USA; echan@dental.ufl.edu
* Correspondence: kesavalu@dental.ufl.edu; Tel.: +1-352-273-6500
† Present address: Department of Neurology, College of Medicine, University of Florida, Gainesville, FL 32610, USA

Supplementary Information:

Table S1. List of upregulated miRNAs at 8-weeks of infection compared to sham-infection.

miRs	Fold Change	*p*-Value
miR-804	1.57	0.009465
miR-690	1.53	0.007031
miR-1224	1.4	0.013375
miR-31	1.37	0.034075
miR-133b	1.36	0.03893
miR-185	1.32	0.000534
miR-19b	1.31	0.000122
miR-22	1.31	0.009126
miR-193	1.29	0.006863
miR-1198	1.27	0.001943
miR-154	1.27	0.04589
miR-152	1.26	0.031135
miR-423-3p	1.26	0.044217
miR-125b-5p	1.24	0.02166
miR-191	1.23	0.013163
miR-107	1.22	0.022686
miR-103	1.21	0.004192
miR-322	1.19	0.019203
miR-30d	1.17	0.042862
miR-301a	1.15	0.041072
miR-15a	1.15	0.046356
miR-30c	1.15	0.049249
miR-30e	1.13	0.00518
miR-151-5p	1.13	0.02108
miR-28	1.13	0.041907
miR-151-3p	1.11	0.030216

Table S2. List of downregulated miRNAs during 8-weeks infection compared to sham-infection.

miRs	Fold Change	*p*-Value
miR-1902	-1.67	0.004943
miR-1937a+miR-1937b	-1.65	0.000118
mmu-let-7f	-1.56	4.04E-05
mmu-let-7c	-1.49	0.000261
mmu-let-7a	-1.48	0.000101
miR-98	-1.46	3.85E-05
miR-127	-1.45	3.04E-05
miR-218	-1.39	0.005713
miR-144	-1.36	0.036176
miR-2133	-1.32	0.034422
miR-720	-1.27	0.001453
miR-29b	-1.25	0.00815
miR-29a	-1.18	0.049705
let-7g	-1.1	0.015144

Table S3. List of upregulated and downregulated miRNAs in *P. gingivalis*-16-weeks infection compared to sham-infection.

	**Upregulated miRNA**	
**miRs**	**Fold Change**	***p*-Value**
miR-30d	1.11	0.046212
miR-103	1.13	0.044262
miR-145	1.14	0.018223
miR-195	1.18	0.028636
miR-24	1.19	0.020169
miR-365	1.22	0.045178
miR-99b	1.24	0.001738
Downregulated miRNA		
miR-302b	-1.12	0.023894

Table S4. Comparison of upregulated miRNAs between 8- weeks and 16-weeks infection.

miRs	Fold change	*p*-value
miR-1937a+miR-1937b	1.54	0.00037449
miR-720	1.35	0.00012091
miR-30b	1.25	0.01897502
miR-1937c	1.27	0.02212542
miR-361	1.3	0.03815099
miR-100	1.12	0.03700248
miR-410	1.19	0.00779958
miR-495	1.23	0.04007289
miR-145	1.22	0.04251919
mmu-let-7f	1.24	0.0456108
miR-26b	1.11	0.04631309

Table S5. Comparison of downregulated miRNAs in *P. gingivalis* infection between 8- and 16-weeks.

miRs	Fold Change	*p*-Value
miR-804	-1.64	0.01060972
miR-m107-1-3p	-1.54	0.02780248
miR-485	-1.54	0.04032774
miR-1942	-1.52	0.03745166
miR-883b-5p	-1.52	0.03901862
miR-669j	-1.52	0.03922487
miR-670	-1.51	0.02803583
miR-1941-5p	-1.5	0.04519783
miR-3474	-1.49	0.03789401
miR-M23-1-5p	-1.49	0.03807786
miR-501-5p	-1.48	0.03058914
miR-1190	-1.48	0.04250021
miR-669i	-1.47	0.03065781
miR-105	-1.47	0.03368492
miR-1898	-1.47	0.04155007
miR-464	-1.47	0.04410116
miR-1903	-1.47	0.04909801
miR-468	-1.47	0.04998101
miR-493	-1.46	0.0308734
miR-1963	-1.45	0.03750653
miR-1197	-1.45	0.04162274
miR-207	-1.45	0.04953777
miR-384-3p	-1.44	0.03375416
miR-1962	-1.43	0.03902775
miR-717	-1.43	0.03916274
miR-708	-1.43	0.04709437
miR-370	-1.43	0.04717626
miR-34b-3p	-1.42	0.03502887
miR-362-3p	-1.4	0.00015343
miR-300	-1.37	0.0451478
miR-154	-1.35	0.03105967
miR-676	-1.34	0.03164852
miR-423-3p	-1.32	0.04086319
miR-329	-1.27	0.02862548
miR-425	-1.27	0.03722069
miR-20a+miR-20b	-1.25	0.01648322
miR-200c	-1.25	0.03575851

Table S6. List of predicted miRNAs and their number of target genes involved in *P. gingivalis*-mediated invasion of epithelial cells.

miRNAs	Fold Change	*p*-Value	# of Genes	Target Genes
miR-31	1.37	0.034075	11	*Pik3r1, Cblb, Cltc, WasI, Arheg26, Dock1, Gab1, Vcl, Met, Cdc42, Arpc5*
miR-133b	1.36	0.03893	6	*Bcar1, Sept8, Was1, Cd2ap, Arpc5*
miR-185	1.32	0.000534	3	*Pxn, Ctnna1, Fn1*
miR-19b	1.31	0.000122	9	*Pik3r3, Cltc, Was1, Src, Actb, Arpc1a, Crk, Itgb1, Arpc5*
miR-22	1.31	0.009126	13	*Cb1, Pik3r1, Pik3r3, Ctnna1, Bcar1, Sept8, Dnm1, Wasf2, Rhoa, Actb, Crk1, Rac1, Cav3*
miR-154	1.27	0.04589	2	*Arpc4, Gab1*
miR-152	1.26	0.031135	11	*Pik3r1, Pik3r3, Cblb, Cltc, Clta, Itga5, Actb, Arpc4, Gab1, Met, Cdc42*
miR-125b-5p	1.24	0.02166	11	*Pik3r3, Bcar1, Sept8, Wasf2, Ptk2, Pik3r2, Itga5, Fn1, Crk, Itgb1, Cds42*
miR-191	1.23	0.013163	1	*Pik3r1*
miR-107	1.22	0.022686	2	Cltc, Rac1
miR-103	1.21	0.004192	2	*Cltc, Rac1*
miR-322	1.19	0.019203	19	*Pik3r3, Ctnna1, Bcar1, Sept8, Dnm1, Cltc, Was1, Dnm2, Sept2, Itga5, Actb, Crk1, Fn1, Elmo2, Vcl, Sept11, Arpc1b, Met, Cdc42*
miR-30d	1.17	0.042862	10	*Pik3r1, Ctnna1, Cblb, Sept8, Was1, Sept2, Rac1, Crk, Itgb1, Pik3cd*
miR-301a	1.15	0.041072	8	*Cblb, Cltc, Was1, Clta, Pik3cb, Arpc1a, Met, Cav2*
miR-15a	1.15	0.046356	9	*Pik3r1, Cltc, Was1, Sept2, Actb, Crk, Cd2ap, Elmo1, Cdc42*
miR-30c	1.15	0.049249	10	*Pik3r1, Ctnna1, Cblb, Sept8, Was1, Sept2, Rac1, Crk, Itgb1, Pik3cd*
miR-30e	1.13	0.00518	10	*Pik3r1, Ctnna1, Cblb, Sept8, Was1, Sept2, Rac1, Crk, Itgb1, Pik3cd*
miR-24	1.19	0.020169	2	*Cblb, Cltc*

Eighteen miRNAs were found to be involved in the bacterial invasion of epithelial cells in mice mandibles infected with P. gingivalis. miR-322 targets and regulate 19 genes that are involved in bacterial invasion of epithelial cells.

Table S7. Distribution of *P. gingivalis* genomic DNA of periodontal bacteria to distal organs.

Monobacterial Infection	Positive Systemic Tissue Samples (n=5 Males and 5 Females)						
	Sex (M/F)	Heart	Lung	Brain	Liver	Kidney	Spleen
P. gingivalis (16-W)	M	0	0	0	0	0	0
	F	1	0	1	0	0	0
